# On the Performance of a Capacitively Coupled Electrical Impedance Tomography Sensor with Different Configurations

**DOI:** 10.3390/s20205787

**Published:** 2020-10-13

**Authors:** Yandan Jiang, Xuekai He, Baoliang Wang, Zhiyao Huang, Manuchehr Soleimani

**Affiliations:** 1 State Key Laboratory of Industrial Control Technology, College of Control Science and Engineering, Zhejiang University, Hangzhou 310027, China; hexuekai@zju.edu.cn (X.H.); wangbl@zju.edu.cn (B.W.); zy_huang@zju.edu.cn (Z.H.); 2 Engineering Tomography Laboratory (ETL), Department of Electronic and Electrical Engineering, University of Bath, Bath BA2 7AY, UK; M.Soleimani@bath.ac.uk

**Keywords:** process tomography, electrical tomography, electrical impedance tomography, capacitively coupled electrical impedance tomography (CCEIT), electrical tomography sensor, shielded configuration

## Abstract

Capacitively coupled electrical impedance tomography (CCEIT) is a new kind of electrical resistance tomography (ERT) which realizes contactless measurement by capacitive coupling and extends traditional resistance measurement to total impedance measurement. This work investigates the performance of a CCEIT sensor with three different configurations, including the unshielded configuration, the shielded configuration A (the CCEIT sensor with the external shield) and the shielded configuration B (the CCEIT sensor with both the external shield and the radial screens). The equivalent circuit models of the measurement electrode pair of the CCEIT sensor with different configurations were developed. Additionally, three CCEIT prototypes corresponding to the three configurations were developed. Both the simulation work and experiments were carried out to compare various aspects of the three CCEIT prototypes, including the sensitivity distribution, the impedance measurement and the practical imaging performance. Simulation results show that shielded configurations improve the overall average sensitivity of the sensitivity distributions. Shielded configuration A contributes to improve the uniformity of the sensitivity distributions, while shielded configuration B reduces the uniformity in most cases. Experimental results show that the shielded configurations have no significant influence on the imaging quality of the real part of impedance measurement, but do make sense in improving the imaging performance of the imaginary part and the amplitude of impedance measurement. However, configuration B (with radial screens) has no significant advantage over configuration A (without radial screens). This work provides an insight into how shielding measures influence the performance of the CCEIT sensor, in addition to playing an important role in shielding unwanted noise and disturbances. The research results can provide a useful reference for further development of CCEIT sensors.

## 1. Introduction

Capacitively coupled electrical impedance tomography (CCEIT) was proposed as a new kind of contactless electrical tomography (ET) technique for the measurement of gas–liquid two-phase flow, by referring to the contactless measurement idea of the capacitively coupled contactless conductivity detection (C^4^D) technique [1,2,3,4]. It takes the two-phase flow as an equivalent impedance and uses the total impedance information (the amplitude, the real part and the imaginary part of the impedance) for contactless imaging [3]. For special cases where the conductive fluid is regarded as an equivalent resistance/conductance and only the real part of the impedance is used, it is termed capacitively coupled electrical resistance tomography (CCERT) [1]. Compared with the conventional electrical resistance tomography (ERT) technique [5,6], the new CCEIT technique can not only avoid the drawbacks of contact measurement (such as electrochemical erosion, polarization and contamination of the electrodes), but also make use of the total impedance information of the gas–liquid two-phase flow [1,2,7,8]. So, it has received wide attention from researchers in the research field of industrial process tomography [1,2,3,4,8,9,10,11]. 

In the research field of ET, sensor configuration is an important research focus [12,13,14,15]. Shielding is usually an essential component in ET sensors to protect the sensor from external electromagnetic interferences. The use of shielding is required to be applied in practical industrial conditions. Related ET studies have provided general shielding measures for reference and the effectiveness of shielding measures in suspension of internal and external interferences is well known [16,17,18,19,20,21,22,23,24,25]. However, as a new technique, CCEIT is still developing. The research is not sufficient. Previous research mainly focuses on the unshielded CCEIT sensors, i.e., shielding structure is not the main focus of CCEIT yet and few studies concerning the shielded structure of CCEIT sensors have been published [2,10,11]. So, more research work on different configurations of CCEIT sensor should be undertaken.

This work aims to study the performance of a 12-electrode CCEIT sensor with different configurations. As the role of shielding in anti-interference is unquestionable, the main focus of this work is not anti-interference performance of different configurations, but how different configurations influence the CCEIT performance, including the sensitivity distribution, the impedance measurement and the practical imaging performance. Two shielded configurations of the CCEIT sensor (A is the sensor with the external shield and B is the sensor with both the external shield and the radial screens) will be investigated and compared with the unshielded configuration of the sensor. The equivalent circuit models of the measurement electrode pair of the CCEIT sensor with the three configurations will be developed, respectively. Simulation work will be completed to study and compare the sensitivity distributions of the three CCEIT sensors. Three prototypes of the three CCEIT sensors will also be developed and experiments will be carried out to evaluate the practical imaging performance of the CCEIT prototypes. 

## 2. Sensor Configurations and Equivalent Circuit Models

In this work, two shielded configurations, which are widely studied and applied in the ET filed [16,17,18,19,20,21,22,23,24,25], are applied to the CCEIT sensor and compared with the unshielded configuration.

### 2.1. The Sensor Configurations

**Figure 1:**
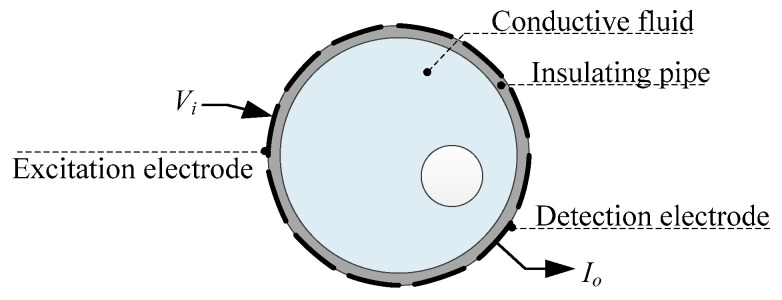
Unshielded configuration: the 12-electrode capacitively coupled electrical impedance tomography (CCEIT) sensor without shielding.

Figure 1 shows the unshielded 12-electrode CCEIT sensor filled with conductive fluid, which includes the insulating pipe and the 12 electrodes. The electrodes are installed equidistantly on the outside of the pipe, which ensures contactless measurement [3].

**Figure 2:**
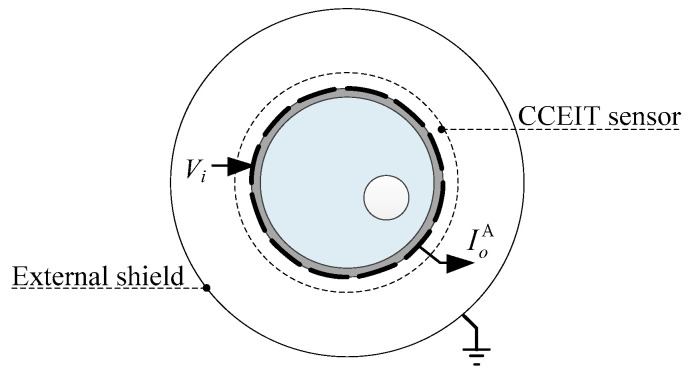
Shielded configuration A: the 12-electrode CCEIT sensor with the external shield.

Figure 2 shows the shielded configuration A, which is the 12-electrode CCEIT sensor with an external shield. The external shield, also termed as external screen, is a very commonly used shielding measure in the ET field to protect the sensor from external interferences [24].

**Figure 3:**
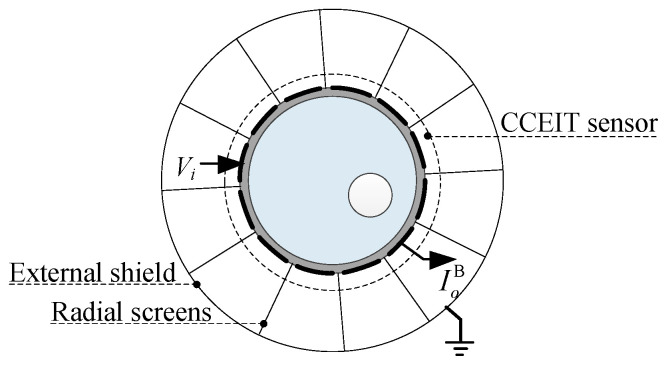
Shielded configuration B: the 12-electrode CCEIT sensor with both the external shield and the radial screens.

Figure 3 shows the shielded configuration B, i.e., the CCEIT sensor with both the external shield and the radial screens. The radial screen, also called radial guard or radial electrode, is an efficient way to avoid the unfavorable stray capacitance formed between electrodes through the air, especially for the adjacent electrodes [25]. So, 12 radial screens are placed in the 12 gaps between the electrodes.

Both of the external shield and radial screens work on the premise of being grounded. 

### 2.2. The Equivalent Circuit Models

#### 2.2.1. The Unshielded Configuration

**Figure 4:**
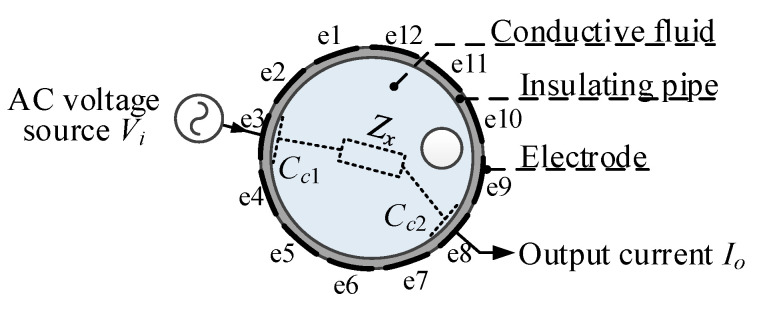
Measurement principle of CCEIT.

**Figure 5:**
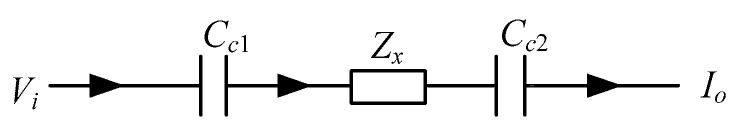
Simplified equivalent circuit model of the measurement electrode pair for the unshielded CCEIT sensor.

Figure 4 shows the measurement principle of the CCEIT. During every measurement, two electrodes will be selected as the measurement electrode pair (one is the excitation electrode and the other is the detection electrode), while other electrodes are at floating potential. As shown in Figure 4, a coupling capacitance will be formed between each electrode and the conductive fluid via the insulating pipe. So, the simplified equivalent circuit model of the measurement electrode pair of the unshielded CCEIT sensor is equivalent to a fluid impedance *Z_x_* in series with two coupling capacitances *C_c_*_1_ and *C_c_*_2_ [1,3], as shown in Figure 5. When an AC voltage signal is applied to the excitation electrode, an output current signal which carries the impedance information of the fluid can be obtained on the detection electrode.

The total impedance measurement *Z_m_* of the electrode pair for the unshielded CCEIT sensor is:

(1) $$Z_{m}=\frac{V_{i}}{I_{0}}=Z_{x}+\frac{1}{j\omega C_{c}}=Z_{x}-j\frac{1}{2\pi fC_{c}}$$

where, *C_c_* is the total capacitance of *C_c_*_1_ and *C_c_*_2_. *ω* = 2*πf* is the angular frequency of the excitation AC voltage source. *f* is the frequency of the AC voltage source.

**Figure 6:**
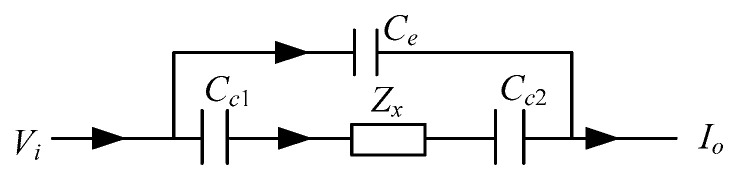
Complete equivalent circuit model of the measurement electrode pair for the unshielded CCEIT sensor.

However, in practical measurement, there also exist stray capacitances formed by the two measurement electrodes, especially when adjacent electrodes are selected as the measurement electrode pair. With two measurement electrodes selected and the excitation voltage applied, the complete equivalent circuit model of the measurement electrode pair for the unshielded CCEIT sensor is illustrated in Figure 6. Where *C_e_* is the external stray capacitance formed by the two electrodes via air. It is obvious that *C_e_* is also in the measurement path and has an influence on the impedance measurement. For a known excitation voltage *V_i_*, the current *I_o_* is determined by the fluid impedance *Z_x_*, the internal coupling capacitances *C_c_*_1_, *C_c_*_2_ and the external stray capacitance *C_e_*.

Then, the total impedance measurement *Z_m_* of the electrode pair for the unshielded CCEIT sensor becomes:

(2) $$Z_{m}=\;\frac{V_{i}}{I_{0}}=\frac{(Z_{x}+\frac{1}{j\omega C_{c}})\frac{1}{j\omega C_{e}}}{Z_{x}+\frac{1}{j\omega C_{c}}+\frac{1}{j\omega C_{e}}}\;=\frac{Z_{x}C_{c}^{2}}{\omega^{2}Z_{x}^{2}C_{c}^{2}C_{e}^{2}+{\left(C_{c}+C_{e}\right)}^{2}}-j\frac{\omega^{2}Z_{x}^{2}C_{c}^{2}C_{e}+(C_{c}+C_{e})}{\omega^{3}Z_{x}^{2}C_{c}^{2}C_{e}^{2}+\omega {\left(C_{c}+C_{e}\right)}^{2}}$$

The electrodes were numbered from e1 to e12 counterclockwise, based on an angle about the center of the sensor (there is an electrode every 30°), as shown in Figure 4. In a whole measurement cycle, electrode e1 is first selected as the excitation electrode and electrode e2–e12 are selected as the detection electrode one by one. Then, electrode e2 is excited and the measurement can be obtained from electrode e3–e12 by turn. This continues until electrode e11 and e12 are selected as the measurement electrode pair. So, there will be 66 independent impedance measurements for the 12-electrode CCEIT sensor.

#### 2.2.2. The Shielded Configuration A (Without Radial Screens)

**Figure 7:**
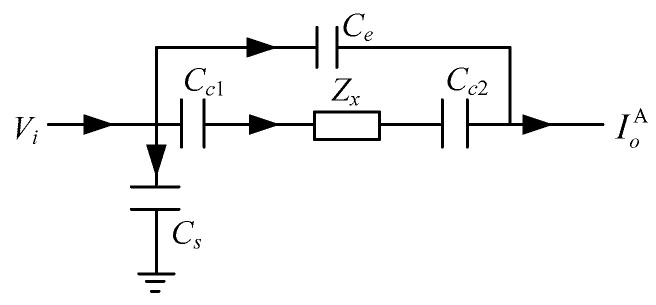
Equivalent circuit model of the measurement electrode pair for the CCEIT sensor with shielded configuration A.

The shielded configuration A is the CCEIT sensor with only the external shield. In this configuration, a stray capacitance will be formed between the electrodes and the shield via air. So, the existence of the external shield will introduce an additional current path for each electrode. Figure 7 shows the corresponding equivalent circuit model of the measurement electrode pair for the CCEIT sensor with shielded configuration A. Compared with the circuit model in Figure 6, the model in Figure 7 has a stray capacitance *C_s_* introduced by the external shield, i.e., *C_s_* is formed by the excitation electrode and the external shield via the air gap between them. The existence of *C_s_* introduces an additional grounded current path to the circuit model. It is necessary to note that because the detection electrode will be connected with the inverting input of an amplifier whose non-inverting input is connected to the ground, i.e., the detection electrode and the grounded shield are equipotential according to the virtual short rule of the amplifier, so there is no stray capacitance formed between the detection electrode and the shield.

The total impedance measurement of the measurement electrode pair for CCEIT sensor with shielded configuration A is:

(3) $$Z_{m}=\frac{V_{i}}{I_{0}^{A}}=\frac{(Z_{x}+\frac{1}{j\omega C_{c}})\frac{1}{j\omega C_{e}}}{Z_{x}+\frac{1}{j\omega C_{c}}+\frac{1}{j\omega C_{e}}}\;=\frac{Z_{x}C_{c}^{2}}{\omega^{2}Z_{x}^{2}C_{c}^{2}C_{e}^{2}+{\left(C_{c}+C_{e}\right)}^{2}}-j\frac{\omega^{2}Z_{x}^{2}C_{c}^{2}C_{e}+(C_{c}+C_{e})}{\omega^{3}Z_{x}^{2}C_{c}^{2}C_{e}^{2}+\omega {\left(C_{c}+C_{e}\right)}^{2}}$$

In this configuration, although the grounded external shield introduces more grounded paths for the signal to go, the measurement path remains unchanged and the detected current *I_o_* contains the same impedance information as that of the unshielded CCEIT sensor. In other words, the composition of the total impedance measurement *Z_m_* of the electrode pair remains the same as that in Equation (2).

#### 2.2.3. The Shielded Configuration B (with Radial Screens)

**Figure 8:**
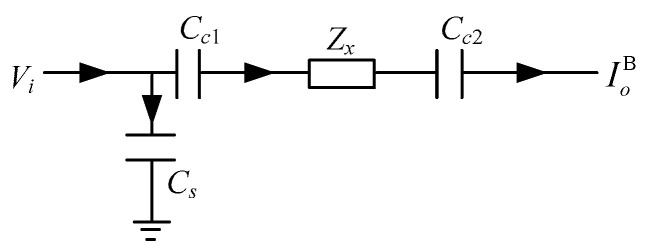
Equivalent circuit model of the measurement electrode pair for the CCEIT sensor with shielded configuration B.

The shielded configuration B is the CCEIT sensor with both the external shield and the radial screens. Figure 8 is the corresponding equivalent circuit model of a measurement electrode pair for the CCEIT sensor with shielded configuration B. It is indicated that the previous external coupling capacitance *C_e_* in the measurement path is eliminated by the radial screens. 

For the CCEIT sensor with the shielded configuration B, the total impedance measurement *Z_m_* of the electrode pair is:

(4) $$Z_{m}=\frac{V_{i}}{I_{0}^{B}}=Z_{x}+\frac{1}{j\omega C_{c}}=Z_{x}-j\frac{1}{2\pi fC_{c}}$$

As can be seen from Figure 6, Figure 7 and Figure 8, the equivalent circuit models of the measurement electrode pair of the CCEIT sensor with different configurations are different.

## 3. Sensitivity Distributions 

### 3.1. Simulation Setup

The sensing area of the CCEIT sensor satisfies the quasi-static electromagnetic field, which is described as [3,26]:

(5) ∇⋅((σ(x,y)+jωε(x,y))∇φ(x,y))=0       (x,y)⊆Ω

where the sensing area is defined as Ω. *σ*(*x*, *y*), *ε*(*x*, *y*) and *φ*(*x*, *y*) are the conductivity, permittivity and potential at the spatial point of coordinates (*x*, *y*) within Ω, respectively.

This work investigates three configurations of CCEIT sensor, so three corresponding groups of boundary conditions can be listed. For the unshielded configuration, the boundary conditions of Equation (5) are:

(6) $$\{\begin{array}{cc} \varphi_{a}(x,y)=V & (x,y)\subseteq \Gamma_{a}\\ \varphi_{b}(x,y)=0 & (x,y)\subseteq \Gamma_{b}\\ \partial \varphi_{c}(x,y)/\partial \vec{n}=0 & \left(x,y)\subseteq \Gamma_{c},(c\neq a,b\right) \end{array}$$

where *V* is the amplitude of the excitation AC voltage source. *a*, *b* and *c* represent the excitation electrode, the detection electrode and the floating electrode, respectively. So, Γ*_a_*, Γ*_b_* and Γ*_c_* represent the spatial regions of the excitation electrode, the detection electrode and the floating electrode. $\vec{n}$ denotes the outward unit normal vector. For the shielded configuration A, the boundary conditions are:

(7) $$\{\begin{array}{cc} \varphi_{a}(x,y)=V & (x,y)\subseteq \Gamma_{a}\\ \varphi_{b}(x,y)=0 & (x,y)\subseteq \Gamma_{b}\\ \partial \varphi_{c}(x,y)/\partial \vec{n}=0 & \left(x,y)\subseteq \Gamma_{c},(c\neq a,b\right)\\ \;\varphi_{s1}(x,y)=0 & (x,y)\subseteq \Gamma_{s1} \end{array}$$

where one more boundary condition of the external shield is added. *s*1 represents the external shield and Γ*_s_*_1_ represents the spatial region of the external shield. For shielded configuration B, the boundary conditions are:

(8) $$\{\begin{array}{cc} \varphi_{a}(x,y)=V & (x,y)\subseteq \Gamma_{a}\\ \varphi_{b}(x,y)=0 & (x,y)\subseteq \Gamma_{b}\\ \partial \varphi_{c}(x,y)/\partial \vec{n}=0 & \left(x,y)\subseteq \Gamma_{c},(c\neq a,b\right)\\ \varphi_{s1}(x,y)=0 & (x,y)\subseteq \Gamma_{s1}\\ \varphi_{s2}(x,y)=0 & (x,y)\subseteq \Gamma_{s2} \end{array}$$

where the boundary condition of the radial screens is added as well. *s*2 represents the radial screens and Γ*_s_*_2_ represents the spatial region of the radial screens. 

### 3.2. Calculation of Sensitivity Matrix

In the research field of process tomography, the sensitivity matrix, which is also known as the Jacobian matrix, is usually obtained by simulation [27]. So, simulation is carried out and the finite element method (FEM) is introduced to obtain the sensitivity matrix *S* = [$s_{mn}$]*_M×N_*. *M* is the number of impedance measurements (*M* = 66) and *N* is the number of elements used to mesh the sensing area. In this work, 32 × 32 square elements are used to mesh the sensing area, so *N* = 1024. The sensitivities (every sensitivity value in the sensitivity matrix) are calculated by joint compilation of software “COMSOL Multiphysics (version 3.4)” and “Matlab (version 2014b)”, i.e., COMSOL is used to implement every finite element calculation, and Matlab is used to implement the loop call of COMSOL and the definition of sensitivity. As the CCEIT sensor focuses on the total impedance measurement, a sensitivity matrix of the real part, a sensitivity matrix of the imaginary part and a sensitivity matrix of the amplitude can be calculated, respectively [3]. For the real part, the sensitivity is defined as:

(9) $$s_{mn}=\frac{R_{mn}-R_{m}^{0}}{R_{m}^{0}}$$

For the imaginary part, the sensitivity is defined as:

(10) $$s_{mn}=\frac{X_{mn}-X_{m}^{0}}{X_{m}^{0}}$$

For the amplitude, the sensitivity is defined as:

(11) $$s_{mn}=\frac{A_{mn}-A_{m}^{0}}{A_{m}^{0}}$$

where *m* = 1, 2, …, *M*, *n* = 1, 2, …, *N*. $R_{m}^{0},\;X_{m}^{0}\;\mathrm{and}\;A_{m}^{0}$ are, respectively, the real part, the imaginary part and the amplitude of the mth impedance measurement when the pipe is full of the liquid phase (*σ* = *σ*_1_, *ε* = *ε*_1_). $R_{mn}$, $X_{mn}$ and $A_{mn}$ are, respectively, the real part, the imaginary part and the amplitude of the *m*th impedance measurement when the *n*th element changes from the liquid phase to the gas phase (*σ* = *σ*_2_, *ε* = *ε*_2_).

### 3.3. Sensitivity Distributions

A sensitivity distribution is a 2D plot of a row in the M*N sensitivity matrix to qualitatively show the sensitivities at different positions in the sensing area. In general, sensitivity distributions are similar for measurement electrode pairs with the same spatial interval between the two electrodes. So, sensitivity distributions of the six typical measurement electrode pairs of the 12-electrode CCEIT sensor (e1–e2, e1–e3, …, e1–e7) are listed in this work for all the three parts of impedance. 

Table 1 shows the typical sensitivity distributions of the real part of impedance measurement obtained by the CCEIT sensors with different configurations, i.e., unshielded, shielded A and shielded B, respectively. In Table 1, the 2D plot presents the sensitivities of the 1024 elements in each of the six typical sensitivity distributions, and the 3D plot represents the sensitivity map consisted of the 1024 sensitivities in the 2D sensing area (here, the sensitivity maps of measurement pair e1–e2, e1–e3 and e1–e7 are listed for demonstration). It can be found that all the sensitivity distributions of the real part of the impedance measurement obtained by the CCEIT sensor with different configurations are not uniform. The highest sensitivities concentrate on the boundary and the lowest sensitivities are situated in the central area. For the unshielded sensor and the shielded sensor with configuration A (without radial screens), all the sensitivities in the typical distributions are positive. While for the shielded sensor with configuration B (with radial screens), negative regions in the typical sensitivity distributions exist, as shown in the 2D plot.

Table 2 shows the plots of the typical sensitivity distributions of the imaginary part of impedance measurement obtained by the CCEIT sensors. The table indicates that the shapes of the six typical distributions of the imaginary part of the impedance measurement obtained by the three sensors are similar. All the sensitivity distributions of the imaginary part are also not uniform, i.e., the sensitivities on the boundary are much higher than those in the central area. Besides, negative regions can be found in the sensitivity distributions of the imaginary part obtained by the CCEIT sensor with shielded configuration B as well.

Table 3 shows the 2D and 3D plots of the typical sensitivity distributions of the amplitude of impedance measurement obtained by the CCEIT sensors. It is necessary to point out that the distributions of the amplitude are similar to those of the imaginary part of the impedance measurement.

To quantitatively evaluate the sensitivity distributions, the average sensitivity, the uniformity coefficient and the fluctuation coefficient are introduced [20,28]. The average sensitivity *S_a_* is defined as the average sensitivity value of all the positive sensitivities in the six typical distributions, which can be described as:

(12) $$S_{a}=\frac{1}{l}\sum_{k=1}^{6}\sum_{n=1}^{N}s_{kn}\;,\;s_{kn}>0$$

where, *k* represents the six typical sensitivity distributions and *k* = 1, 2, …, 6. *l* is the total number of positive sensitivities in the six distributions. 

The uniformity coefficient *S_u_* is the average standard deviation of the six distributions over the average sensitivity. It is expressed as:

(13) $$S_{u}=\frac{1}{6}\sum_{k=1}^{6}\frac{\nu_{k}}{S_{ak}}$$

where, *ν_k_* and *S_ak_* are, respectively, the standard deviation and the average sensitivity of the sensitivities in the *k*th typical sensitivity distribution. 

The fluctuation coefficient *S_f_* is the average gap between the sum of 50 maximum sensitivities and the sum of 50 minimum sensitivities of the six distributions. It is expressed as:

(14) $$S_{f}=\frac{1}{6}\sum_{k=1}^{6}(S_{k}^{\max50}-S_{k}^{\min50})$$

where $S_{k}^{\max50}$ and $S_{k}^{\min50}$ are, respectively, the sum of 50 maximum sensitivities and the sum of 50 minimum sensitivities of the *k*th typical sensitivity distribution.

The average sensitivity means the overall sensitivity of the sensor to the small changes in the sensing area, and the uniformity coefficient and the fluctuation coefficient measure the overall differences between the sensitivities in the sensitivity field. So, higher average sensitivity and lower uniformity coefficients are preferred.

Table 4 shows the three sensitivity indexes concerning the typical sensitivity distributions of the real part, the imaginary part and the amplitude of impedance measurement obtained by the CCEIT sensor with different configurations. It can be found that the sensitivity distributions of the CCEIT sensor with the shielded configurations overall have a higher average sensitivity than the unshielded configurations. Shielded configuration B helps improve the uniformity of sensitivity distribution a little bit when compared with the unshielded configuration. However, the CCEIT with shielded configuration B has the biggest gap between the maximum sensitivities and the minimum sensitivities, which results from the negative sensitivities introduced by the radial screens to the typical sensitivity distributions of the real part of impedance measurement, as can be seen from Table 1, Table 2 and Table 3. For the imaginary part and the amplitude of impedance measurement, the radial screens introduce more negative sensitivities. Negative sensitivity region is usually not desirable because it makes little contribution to the image reconstruction.

## 4. Imaging Performance

### 4.1. Experimental Setup

A practical experiment was carried out to illustrate the influence of shielding measures on practical imaging performance of the CCEIT sensor. Three CCEIT prototypes were developed with the three different configurations.

**Figure 9:**
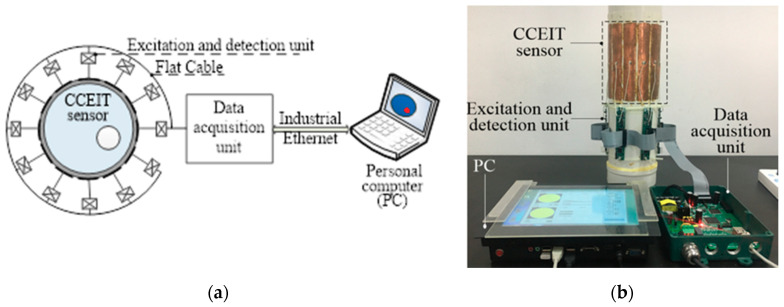
The CCEIT prototype: (**a**) Construction. (**b**) Photo.

As shown in Figure 9, taking the unshielded CCEIT prototype as an example, the CCEIT prototype includes a 12-electrode CCEIT sensor, 12 excitation and detection units, a data acquisition unit and a personal computer (PC). Each electrode is connected with an excitation and detection unit, which mainly implements electrode selection based on the control signal from the data acquisition unit, and implements current-to-voltage conversion (the I/V converter) of the detection signal. 

**Figure 10:**
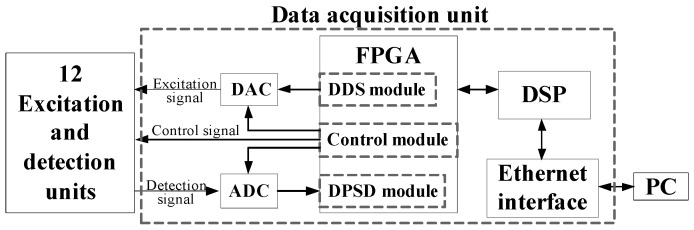
The data acquisition unit. DAC: digital-to-analog converter; ADC: analog-to-digital converter; DSP: digital signal processor; FPGA: field programmable gate array; DSS: direct digital synthesizer; DPSD: digital phase-sensitive demodulation; PC: personal computer.

Figure 10 shows the construction of the data acquisition unit, which consists of the digital signal processor (DSP), the field programmable gate array (FPGA), the digital-to-analog converter (DAC), the analog-to-digital converter (ADC) and the Ethernet interface. DSP controls the whole measurement process. FPGA includes the direct digital synthesizer (DDS) module, the control module and the digital phase-sensitive demodulation (DPSD) module. The DDS and DAC together can implement the generation of the excitation AC voltage signal. The control module controls the DAC, the ADC and the 12 excitation and detection units. The DPSD module is used to demodulate the sampled detection signal and calculate the interested impedance measurements (i.e., the real part, the imaginary part and the amplitude). The Ethernet interface transmits the results to the PC for image reconstruction.

**Figure 11:**
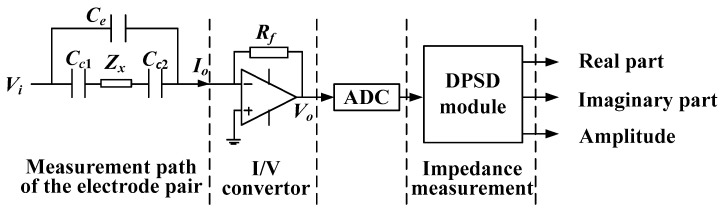
The impedance measurement process of a measurement electrode pair of the CCEIT sensor.

Figure 11 shows the impedance measurement process of a measurement electrode pair of the CCEIT sensor. When an AC voltage source *V_i_* is applied to the excitation electrode, an output current signal *I*_0_ flows through the measurement path of the electrode pair (take the unshielded sensor as an example) can be detected on the detection electrode. Then the current signal *I*_0_ is converted to an output voltage *V*_0_ by the I/V convertor in the excitation and detection unit. Then, the voltage signal is sampled by the ADC and then sent to the DPSD module. Finally, with the operation of the DPSD, impedance measurements including the real part, the imaginary part and the amplitude of the impedance are obtained.

**Figure 12:**
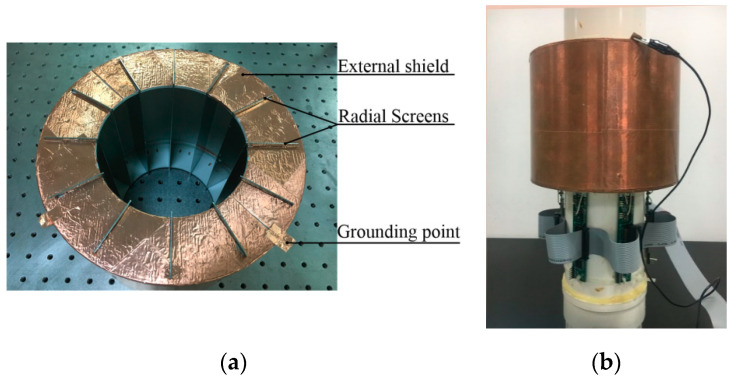
Photo: (**a**) Shields; (**b**) Shielded CCEIT.

In the developed CCEIT prototypes, the outer diameter of the insulating pipe was 110 mm. The material of the pipe is polyvinyl chloride (PVC) and the thickness of the pipe wall was 2 mm. The length and width of the electrodes were 150 mm and 24 mm (i.e., the electrode angle is 25°). The voltage and frequency of the excitation signal were 3.3 V and 500 kHz, respectively. An external shield with 12 detachable radial screens was developed, as shown in Figure 12a. The outer diameter and the height of the external shield were 200 mm and 160 mm, respectively. The external shield was produced by 3D print and was covered by copper sheet. The 12 corresponding radial screens were made of steel sheet. Figure 12b shows a photo of the shielded CCEIT prototype. 

### 4.2. Impedance Measurement

With the three CCEIT prototypes, the influence of shielding measures on the impedance measurement is investigated first. Here, the impedance measurement is implemented when the sensing area is filled with water and the real part of impedance measurement is taken as an example. 

**Figure 13:**
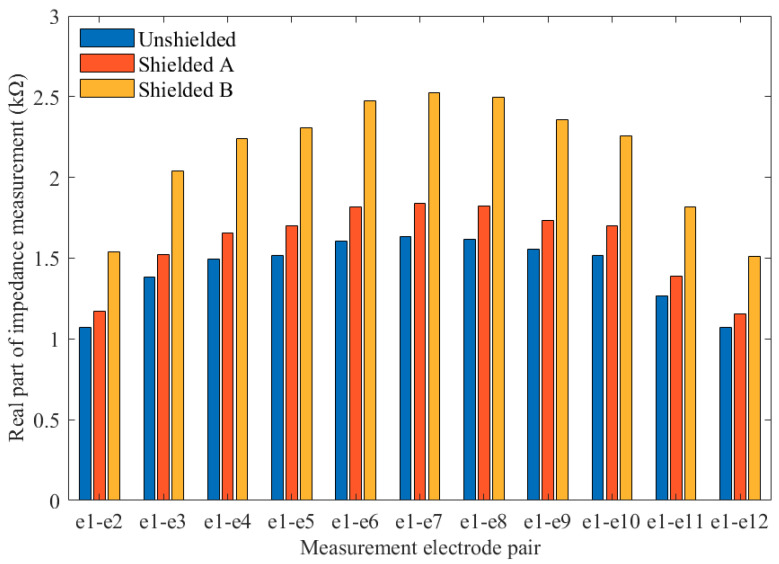
The real parts of 12 impedance measurements obtained by the three CCEIT prototypes.

Figure 13 shows the real parts of 12 impedance measurements obtained by the three CCEIT prototypes, i.e., with unshielded configuration, with shielded configuration A and with shielded configuration B, respectively. Obviously, the impedance measurement increases after the shielding measures are added. Compared with shielded configuration A, configuration B results in a more significant change in impedance measurements. The answer to this increase in impedance measurement can be found in Figure 7 and Figure 8. For both the two shielded configurations, there exists an additional grounded current path formed by the stray capacitance (i.e., *C_s_*) between the excitation electrode and the grounded shields. That means a smaller current signal will be detected on the detection electrode, so the increase in impedance measurement is reasonable.

Table 5 shows the impedance ratios of the three prototypes. For a specified sensor, the impedance ratio is the ratio of the maximum typical measurement to the minimum typical measurement. Take the real part measurement as an example again, the impedance ratio is defined as:

(15) $$\eta =\frac{R_{e1–e7}}{R_{e1–e2}}$$

where *R*_e1–e7_ is the maximum typical real part measurement of impedance, i.e., the measurement obtained when the opposite electrodes e1 and e7 are selected as the measurement electrode pair. *R*_e1–e2_ is the minimum typical real part measurement of impedance, i.e., the measurement obtained when the adjacent electrodes e1 and e2 are selected as the measurement electrode pair.

Larger impedance ratio means higher requirement for the design of subsequent data acquisition circuit because the measurement signal range will be wider. According to the impedance ratios of the real part measurement in Table 5, the external shield will make the impedance ratio slightly larger, and introducing the radial screens make this ratio further larger. 

### 4.3. Imaging Results

Image reconstruction is to obtain the phase distribution image based on the projections and the sensitivity matrix [29]. It can be described as:

(16) P=SG

where *P* = [*p*_1_, *p*_2_, …, *p_m_*, …, *p_M_*]^T^ is the projection vector. *S* is the sensitivity matrix described in Section 3.2. *G* = [*g*_1_, *g*_2_, …, *g_n_*, …, *g_N_*]^T^ is the image vector which reflects the phase distribution. For the real part of the impedance measurement, *p_m_* is defined as:

(17) $$p_{m}=\frac{R_{m}-R_{m}^{0}}{R_{m}^{0}}$$

For the imaginary part of the impedance measurement, *p_m_* is defined as:

(18) $$p_{m}=\frac{X_{m}-X_{m}^{0}}{X_{m}^{0}}$$

For the amplitude of the impedance measurement, *p_m_* is defined as:

(19) $$p_{m}=\frac{A_{m}-A_{m}^{0}}{A_{m}^{0}}$$

where $R_{m}^{0},\;X_{m}^{0}\;\mathrm{and}\;A_{m}^{0}$ are, respectively, the real part, the imaginary part and the amplitude of the mth impedance measurement when the pipe is full of the liquid phase. $R_{m}$, $X_{m}$ and $A_{m}$ are, respectively, the real part, the imaginary part and the amplitude of the mth impedance measurement under the practical phase distribution. 

For each part of the impedance measurement (the real part, the imaginary part and the amplitude), an image *G* can be obtained as the corresponding projection vector *P* and sensitivity matrix *S* are known. In this work, the image is reconstructed by back projecting the boundary projections on the sensitivity matrix, which is known as the linear back projection (LBP) algorithm [24]. According to the LBP algorithm, the phase distribution image *G* = [*g*_1_, *g*_2_, …, *g_n_*, …, *g_N_*]^T^ can be reconstructed as:

(20) $$g_{n}=\frac{\sum_{m=1}^{M}p_{m}s_{mn}}{\sum_{m=1}^{M}s_{mn}}$$

**Figure 14:**
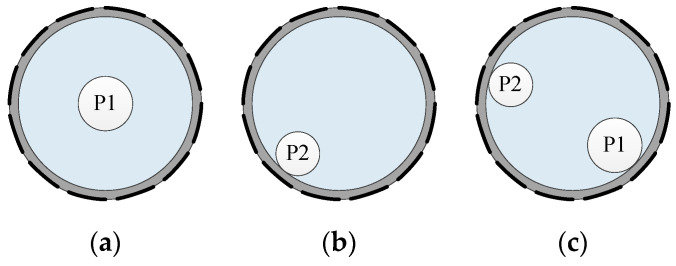
Phase distribution setups: (**a**) S1; (**b**) S2; (**c**) S3.

Three phase distribution setups were tested by taking the tap water (*σ* = 0.025 S/m, *ε* = 80) as the continuous phase and the plastic rods (*σ* = 0 S/m, *ε* = 3) as the disperse phase. The material of the plastic rods is polyethylene (PE). The diameters of plastic rod P1 and P2 were 29.5 mm and 25.5 mm, respectively. The two plastic rods have the same length of 350 mm. Figure 14 shows the distribution setups S1–S3.

Table 6 show the imaging results of phase distribution setup S1 with the real part, the imaginary part and the amplitude of impedance measurements obtained by the three CCEIT prototypes, respectively.

Table 7 shows the imaging results of phase distribution setup S2 with the real part, the imaginary part and the amplitude of impedance measurements obtained by the three CCEIT prototypes, respectively.

Table 8 shows the imaging results of phase distribution setup S3 with the real part, the imaginary part and the amplitude of the impedance measurements obtained by the three CCEIT prototypes, respectively.

To quantitatively evaluate the imaging performance of the three CCEIT prototypes, the relative image error *E_r_* is introduced, which can be described as

(21) $$E_{r}=\sqrt{\frac{\sum_{n=1}^{N}{\left(g_{n}-g_{n}^{0}\right)}^{2}}{\sum_{n=1}^{N}{\left(g_{n}^{0}\right)}^{2}}}$$

where *g_n_* is the grey level of the *n*th element in the reconstructed image and *g_n_*^0^ is the grey level of the *n*th element in the practical distribution image. 

Table 9 shows the imaging performance index of the three CCEIT prototypes.

According to the imaging results of the three CCEIT prototypes, the imaging performance of CCEIT with different configurations is different. Although the three CCEIT prototypes provide comparable images for the real part of the impedance measurement, both the two shielded configurations can effectively improve the imaging performance of CCEIT for the imaginary part and the amplitude of impedance measurement. That is mainly reflected in the distribution setup S2 and S3, as shown in Table 7 and Table 8. Besides, it is found that the images reconstructed by the two shielded CCEIT prototypes are comparable for the imaginary part and the prototype with shielded configuration A even has better imaging performance for the amplitude than that with shielded configuration B, which means introducing additional radial screens to the external shield makes little sense. From this aspect, radial screens are not recommended because they increase the installation cost and complexity but show no advantage according to the current research results.

## 5. Conclusions

This work studies the performance of a 12-electrode CCEIT sensor with three different configurations, including one unshielded configuration and two shielded configurations (configuration A with the external shield and configuration B with both the external shield and the radial screens). With the three configurations, three equivalent circuit models of the measurement electrode pair and three corresponding CCEIT prototypes were developed, respectively. Simulations and experiments were carried out to investigate and compare various aspects of the three CCEIT prototypes, including the sensitivity distributions, the impedance measurements and the practical imaging performance. Based on the current research results, the following conclusions can be obtained:

1. The shielding measures do make a difference in the sensitivity distributions of the CCEIT sensor. According to the average sensitivities of the typical sensitivity distributions obtained by the three CCEIT prototypes, it is indicated that the shielded CCEIT sensors have a higher overall sensitivity than the unshielded CCEIT sensor.
2. Although all the sensitivity distributions of the shielded CCEIT sensors are still not uniform, the external shield has shown value in improving the uniformity of the sensitivity field. However, the radial screens will introduce many regions of negative sensitivities, so shielded configuration with radial screens will reduce the uniformity of sensitivity distribution in most cases.
3. According to the practical imaging results obtained by the LBP algorithm, it is found that all the reconstructed images of the three CCEIT sensors are consistent with the actual distributions. This verifies the effectiveness of the three developed CCEIT prototypes.
4. Imaging results using the real part of the impedance measurement show that the shielding measures have little influence on the imaging quality. While when the imaginary part and the amplitude of impedance measurement are used, it is found that the shielded configurations help to improve the imaging performance of CCEIT. Comparing the current imaging results of the two shielded CCEIT prototypes, images obtained by the shielded prototype with radial screens show no advantage over those obtained by the shielded prototype without radial screens, which means introducing additional radial screens to the external shield is not necessarily helpful for imaging.

For challenging imaging situations where high accuracy is needed, shielding is an essential part of the sensor and will have important role. As a new proposed ET technique, there is a lack of research on the shielded structure of CCEIT. This work provides an insight into how shielding influences the performance of the CCEIT sensor, in addition to playing an important role in shielding unwanted noise and disturbances. New knowledge and experience on the shielded CCEIT sensor are obtained. The research results can provide useful reference for further development of CCEIT sensors.

## Figures and Tables

**Table 1:** Typical sensitivity distributions of the real part of impedance measurement obtained by CCEIT sensors.

Configuration	2D Plot	3D Plot			
		e1–e2	e1–e3	…	e1–e7
Unshielded	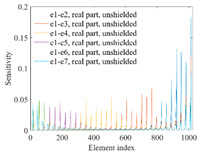	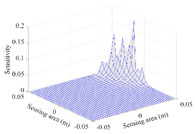	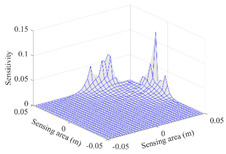	…	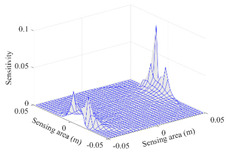
Shielded A	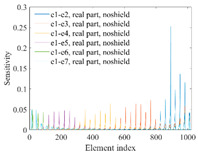	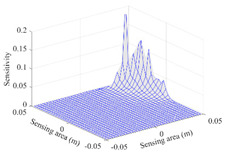	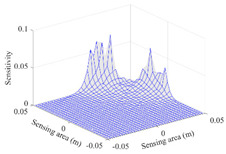	…	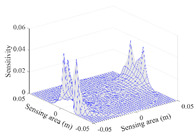
Shielded B	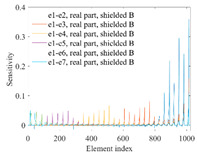	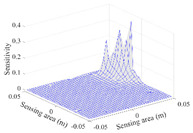	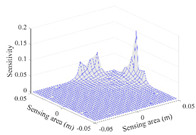	…	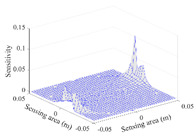

**Table 2:** Typical sensitivity distributions of the imaginary part of impedance measurement obtained by CCEIT sensors.

Configuration	2D Plot	3D Plot			
		e1–e2	e1–e3	…	e1–e7
Unshielded	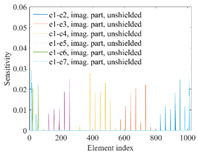	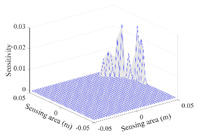	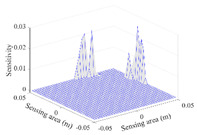	…	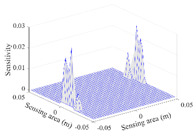
Shielded A	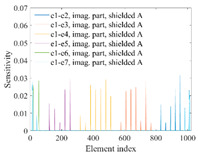	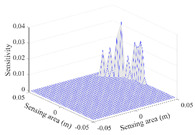	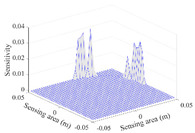	…	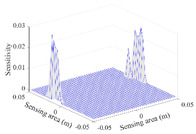
Shielded B	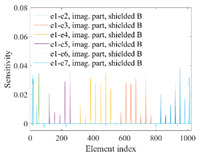	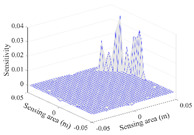	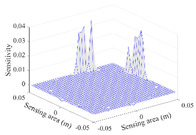	…	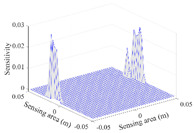

**Table 3:** Typical sensitivity distributions of the amplitude of impedance measurement obtained by CCEIT sensors.

Configuration	2D Plot	3D Plot			
		e1–e2	e1–e3	…	e1–e7
Unshielded	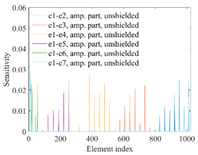	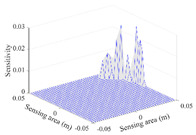	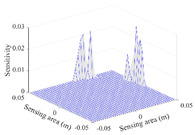	…	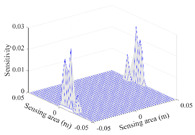
Shielded A	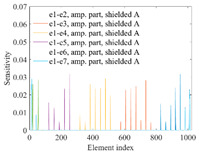	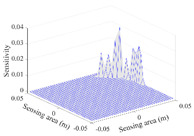	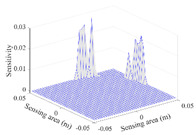	…	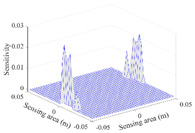
Shielded B	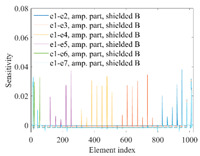	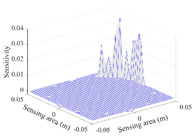	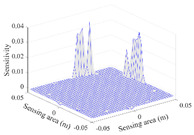	…	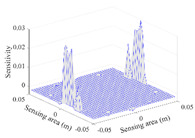

**Table 4:** Indexes of the typical sensitivity distributions.

Configuration	The Real Part			The Imaginary Part			The Amplitude		
	*S_a_*	*S_u_*	*S_f_*	*S_a_*	*S_u_*	*S_f_*	*S_a_*	*S_u_*	*S_f_*
Unshielded	0.00240	2.78434	1.23761	0.00021	8.98697	0.20766	0.00021	8.90382	0.20854
Shielded A	0.00236	2.72383	1.24445	0.00027	8.66250	0.26497	0.00024	8.92217	0.23670
Shielded B	0.00252	3.59691	1.72335	0.00028	9.69043	0.32962	0.00069	6.26585	0.36717

**Table 5:** Impedance ratios (real part) of the three CCEIT prototypes.

CCEIT Sensor	Impedance Ratio
Unshielded	1.5182
Shielded A	1.5682
Shielded B	1.5977

**Table 6:** Imaging results of S1 obtained by the CCEIT prototypes.

Configuration	The Real Part	The Imaginary Part	The Amplitude	
Unshielded	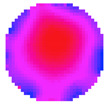	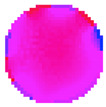	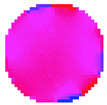	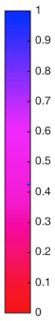
Shielded A	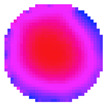	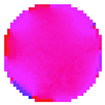	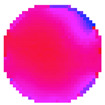	
Shielded B	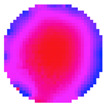	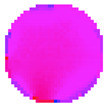	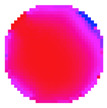	

**Table 7:** Imaging results of S2 obtained by the CCEIT prototypes.

Configuration	The Real Part	The Imaginary Part	The Amplitude	
Unshielded	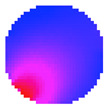	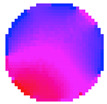	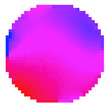	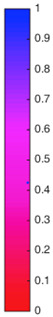
Shielded A	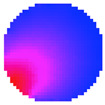	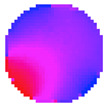	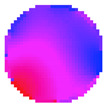	
Shielded B	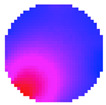	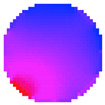	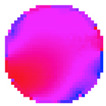	

**Table 8:** Imaging results of S3 obtained by the CCEIT prototypes.

Configuration	The Real Part	The Imaginary Part	The Amplitude	
Unshielded	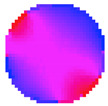	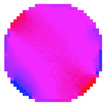	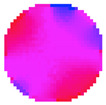	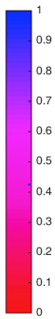
Shielded A	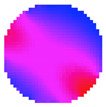	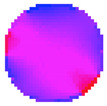	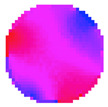	
Shielded B	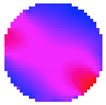	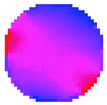	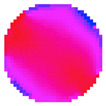	

**Table 9:** Imaging performance index *E_r_* of the three CCEIT prototypes.

Configuration	The Real Part			The Imaginary Part			The Amplitude		
	S1	S2	S3	S1	S2	S3	S1	S2	S3
Unshielded	0.5708	0.2259	0.3757	0.5211	0.3789	0.4706	0.7309	0.6593	0.6896
Shielded A	0.5300	0.2708	0.3568	0.4879	0.3344	0.3674	0.6815	0.3537	0.5990
Shielded B	0.5679	0.3078	0.3436	0.5481	0.2727	0.3471	0.7058	0.6516	0.7146
